# Electrical and Optical Characterization of CsPbCl_3_ Films around the High-Temperature Phase Transitions

**DOI:** 10.3390/nano12030570

**Published:** 2022-02-07

**Authors:** Mara Bruzzi, Matteo Latino, Naomi Falsini, Nicola Calisi, Anna Vinattieri

**Affiliations:** 1 Department of Physics and Astronomy, University of Florence, Via G. Sansone 1, 50019 Sesto Fiorentino, Italy; naomi.falsini@unifi.it (N.F.); anna.vinattieri@unifi.it (A.V.); 2 Istituto Nazionale di Fisica Nucleare Sezione di Firenze, Via G. Sansone 1, 50019 Sesto Fiorentino, Italy; 3 Department of Information Engineering, University of Florence, Via S. Marta 3, 50139 Florence, Italy; matteo.latino@stud.unifi.it; 4 Nuclear Safety, Security and Sustainability Division, Fusion and Technology for Nuclear Safety and Security Department, Italian National Agency for New Technologies, Energy and Sustainable Economic Development (ENEA), Via Martiri di Monte Sole 4, 40129 Bologna, Italy; 5 Department of Industrial Engineering (DIEF), University of Florence, Via S. Marta 3, 50139 Florence, Italy; nicola.calisi@unifi.it; 6 European Laboratory for Non-Linear Spectroscopy (LENS), Via N. Carrara 1, 50019 Sesto Fiorentino, Italy

**Keywords:** halide perovskites, thin films, phase transitions, TSC

## Abstract

Large-area CsPbCl_3_ films in the range 0.1–1.5 μm have been grown by radio frequency (RF)-magnetron sputtering on glass substrates by means of a one-step procedure. Three structural phase transitions have been detected, which are associated with hysteresis behavior in the electrical current when measured as a function of temperature in the range 295–330 K. Similarly, photoluminescence (PL) experiments in the same temperature range bring evidence of a non-monotonic shift of the PL peak. Detailed electrical characterizations evidenced how phase transitions are not influencing detrimentally the electrical transport properties of the films. In particular, the activation energy (0.6–0.8 eV) extracted from the temperature-dependent film resistivity does not appear to be correlated with phase changes. A non-linear trend of the photoconductivity response as a function of a ultra violet (UV) 365 nm light emitting diode (LED) power has been interpreted considering the presence of an exponential tail of intragap defects. Thermally stimulated currents after exposure with the same LED measured from room temperature up to 370 K showed no evidence of trapping effects due to intragap states on the electrical transport properties at room temperature of the films. As a consequence, measured photocurrents at room temperature appear to be well reproducible and stable in time, which are attractive features for possible future applications in photodetection.

## 1. Introduction

Metal halide perovskites are semiconducting materials with a general formula AMX_3_ where A is Cs^+^, CH_3_NH_3_^+^ methylammonium, M are metal cations (Pb^2+^ or Sn^2+^), and X are halide anions (I^−^, Br^−^, or Cl^−^). This material class has received enormous attention in the scientific community recently due to breakthroughs in optoelectronic applications, mainly in photovoltaics [1,2], photodetectors [3,4,5,6,7], light emission [8,9], and lasing [10]. All-inorganic cesium lead halides (CsPbX_3_, X = Cl, Br, I), having the same prominent optoelectronic properties, such as long carrier diffusion distance, huge absorption coefficient, high photoluminescence quantum yield (PLQY), and narrow emission band as the hybrid organic–inorganic perovskites, show an improved stability and lifetime under operation and in ambient conditions. Recently, CsPbCl_3_ with a band gap ≈ 3 eV at 300 K has driven a lot of attention for the realization of efficient light emitters in the blue spectral range, instead of GaN and AlGaN and, in combination with a converting phosphor, for white LEDs [11]. Unlike other inorganic lead halide perovskites, which are commonly realized by solution-based techniques, CsPbCl_3_ synthesis is more difficult; it has been recently reported that vapor phase deposition, in particular RF magnetron sputtering, allows for the deposition of nanometric films with a good degree of homogeneity on a large scale and state of the art optical quality [12,13]. A common feature of CsPbX_3_ perovskites is the presence of structural phase transitions from the low symmetry lattice (monoclinic) at low temperature to the high symmetric lattice (cubic) at high temperature (T). Of course, the values of T at which transition from monoclinic-tetragonal-cubic lattice occurs depend on the specific kind of perovskite. For CsPbCl_3_ bulk samples, a few phase transitions have been reported by several authors using different techniques (Raman spectra, X-ray diffraction studies, heat flow) in the temperature range 310–320 K [14,15,16,17]. In this work, we investigate the correlation between the structural phase transition and the optical/electrical characteristics of CsPbCl_3_ thin films realized by RF magnetron sputtering. The focus of the research presented here is to bring evidence of the role of these phase transitions on the carrier transport and radiative recombination. Given the interest of device realization using CsPbCl_3_ as active material, in particular for LEDs, the relevance of this study appears clear, being the device operating around room temperature.

## 2. Materials and Methods

CsPbCl_3_ films in the range 0.1–1.5 μm have been deposited by an RF-magnetron sputtering technique on glass substrates by means of a one-step procedure: the sputtering target (a disk of several cm in diameter) was realized by pressing (11.5 MPa) for 24 h at 423 K the powder obtained by milling the precursor salts (CsCl and PbCl_2_) in an equal molar ratio. The deposition was performed at room temperature by using a Korvus HEX system (Korvus Technology Ltd., Newington, UK) equipped with an RF source at 13.56 MHz with a power of 20 W and an argon gas flow of 20 sccm. The dynamic working pressure was 2 × 10^−6^ atm and the deposition rate was between 5 and 7 × 10^−2^ nms^−1^. The samples were fully characterized concerning morphology, structure, and stoichiometry, as extensively described in [12,18,19].

Samples were investigated by photoluminescence (PL) spectroscopy in the temperature range 300–330 K; the sample was kept on an XY translation (Physik Instrumente, Karlsruhe, Germany) stage, and PL was excited by means of a pulsed laser emitting at 266 nm (well above the energy band gap of CsPbCl_3_) with a repetition rate of 20 kHz delivering 300 ps long pulses. The excitation spot was of the order of ≈0.5 mm^2^, and the luminescence was collected by a home-made confocal microscope setup equipped with a Mitutoyo (Mitutoyo, Kawasaki, Japan) long working distance 50× objective (NA = 0.42). The luminescence was spectrally dispersed and detected using a flat field spectrometer (Andor Kymera 193i (Andor-Oxford Instruments, Belfast, North Ireland) equipped with a 1200 gr/mm grating blazed at 500 nm and a CCD (Andor iDus, 1024 × 255 pixel 26 × 26 µm^2^, (Korvus Technology Ltd., Newington, UK) connected to a computer. The spatial resolution of the system is about 2 µm, while the spectral resolution is about 2 meV in the spectral range of interest. By means of the XY translation stage, we could span the whole excited area to compare the photo luminescence (PL) spectra in different microscopic regions to assess the sample homogeneity.

Room temperature (RT) transmittance spectra have been acquired, shining the sample by means of an LED with central wavelength at 415 nm with 12 nm FWHM (Full Width at Half Maximum). The transmitted light was detected by means of the detection system (monochromator and CCD) previously described.

In the experimental setup for electrical measurements, the sample deposited on glass is placed on a copper sample-holder equipped with a 20 Ω conducting wire powered by a TTi QL564P power supply (Thurlby Thandar Instruments, Glebe Rd, Huntingdon, Cambridgeshire, PE29 7DR, UK). The temperature is controlled by two Pt100 temperature sensors (Innovative Sensor Technology, Las Vegas, NV, USA), one placed directly on the film and the other one on a glass of the same thickness placed in the neighbor of the sample. The two Pt100 resistances are read out with four-wires probes respectively by two electrometers, Keithley 2001 and 199 (Keithley Instruments LLC, Solon, OH, USA). A two-terminal probe, model 844 by ETS (Electro-Tech Systems, Inc. Perkasie, PA, USA), is used to electrically connect the sample. The 844 ETS probe is especially designed to meet the ANSI/ESDA STM11.13 requirements (ESD Electrostatic Discharge Association Inc., NY, USA) for testing the electrical resistance of thin films deposited on large areas. It is equipped with gold-plated, spring-loaded flat pins with conductive rubber electrodes at contact to provide good adhesion without mechanically deteriorating the film. It has been placed directly on the CsPbCl_3_ film and connected to the Keithley 6517 high-resistance source/electrometer (Keithley Instruments LLC, Solon, OH, USA). This instrument is able to supply external voltages up to 1 kV and monitor current in the range 10 fA–10 mA, using 10–20 ms integration times. Considering the whole electrical system made of probe, source/meter, and coaxial cabling, our setup is characterized by a 10^−14^ A resolution in the current read-out. A customized MATLAB code (Matlab2020b, Mathworks, The MathWorks Inc., Natick, MA, USA) is used to measure the current, to apply the external voltage, and to control the temperature during the measurements.

The electrical resistivity of a set of samples with thickness *t* = 100–200–500–1300–1500 nm has been determined by current–voltage (I–V) measurements carried out in dark at room temperature. An important issue when studying the electrical performance of halide perovskite samples is the stability in time of the current at constant applied voltages [20]. In fact, polarization effects may appear, giving rise to a decay of the current during time and hysteretic behavior of the I–V characteristics. Thus, the I–V measurement has been carried out in a cycle in the range ±500 V by applying different external voltages with increasing/decreasing steps (±100 V). At each step, the current has been monitored over a long time window (250 s) to detect possible instabilities due to polarization.

In a second experiment, the photocurrent of the sample has been measured at room temperature during illumination with a 365 nm (UV) LED. The LED light has been repeatedly flashed on the sample to study the possible occurrence of transient effects when the light is switched on/off and to evaluate the photocurrent in stationary conditions. Photocurrent plateau values have been studied as a function of the LED illumination power in the range 0.5–25 mW.

In a third experiment, the current has been measured at a fixed applied voltage as a function of the temperature (I–T) in the range 295–330 K at constant heating/cooling rates in the range 0.01–0.1 K/s. I–T curves have been measured both in dark and under UV LED illumination at different power and applied voltages, so to evidence if phase transitions may influence the electrical conductivity of the films, to verify that the current is thermally activated, and finally to evaluate the activation energy E_A_ in different illumination conditions.

Finally, a thermally stimulated current experiment has been carried out. Here, the sample, initially primed at a constant applied voltage with a long UV LED pulse at room temperature, has been first heated up to 370 K with a constant heating rate and then cooled down to room temperature again. The test has been performed in order to verify if the electrical response of the film is influenced by charge carriers capture/emission from deep energy levels due to lattice defects and eventually estimate trap parameters as energy level distance from conduction/valence band edges as well as capture the cross-section and concentration.

## 3. Experimental Results

### 3.1. Room-Temperature Electrical Characterization

**Figure 1:**
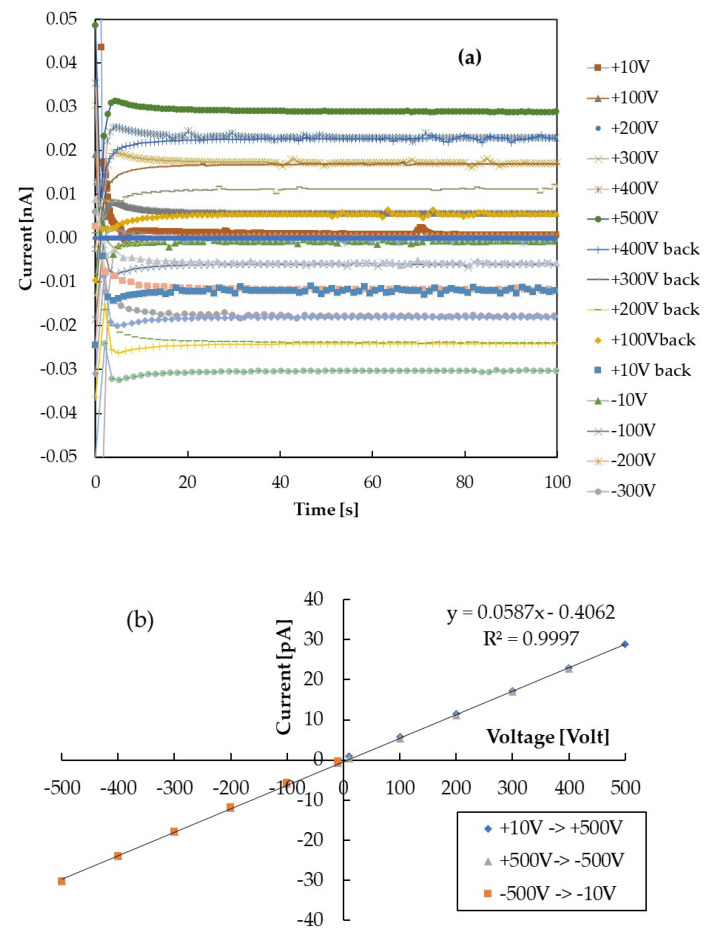
(**a**) Current as a function of time measured with a 200 nm-thick sample at room temperature by applying different external voltages in a cycle from 10 to 500 V, then back down to −500 V and finally to −10 V. (**b**) I–V characteristics obtained from (**a**) by averaging the current measured as a function of time on a time window from 20 to 250 s.

**Figure 2:**
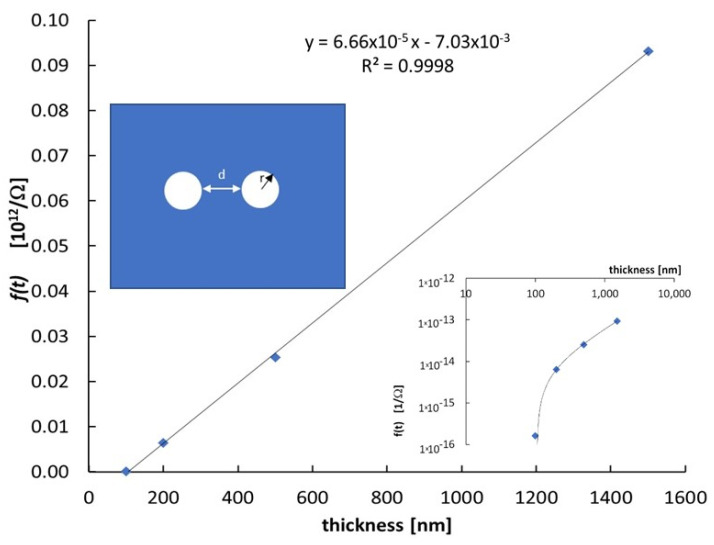
The function $f=\frac{ln\;2\;}{2\pi R}$ with R electrical resistance of the film, is plotted as a function of the film thickness t to determine the electrical conductivity and to test the homogeneity of the semiconductor material produced with different deposition times with the same growth parameters. Inset shows the logarithmic plot to evidence that the linear behavior holds on several decades. Electrode geometry is also sketched at bottom right.

First, we have investigated the stability of the dark current at constant applied voltage and temperature. In Figure 1a, we show the current as a function of time for a 200 nm-thick CsPbCl_3_ film at T = 295 K. The current vs. time trend is reported for different applied voltages obtained by changing the polarization condition in a cycle from 10 to 500 V, then back down to −500 V, and finally to −10 V. Figure 1b shows the I–V characteristics obtained by averaging the current measured as a function of time on time windows from 20 up to 250 s, in the range ±500 V. All the I–V characteristics measured with films of different thicknesses have been found to be linear as in Figure 1b. The resistance R of each film has been calculated as the slope of the corresponding V(I) function. Considering the geometry of the probe (see Figure 2), made of two circular electrodes with *r* = 1.5 mm distant *d = 2r*, placed on a film with *t* << *r* and linear size *L* >> *d*, the relationship between resistance and electrical conductivity *σ* is:

(1) $$R=\int \frac{dh}{\sigma S}=\int_{r}^{2r}\frac{dr}{2\pi r\sigma t}=\frac{ln2}{2\pi \sigma \;}\frac{1}{t}$$

The resistance *R* measured at each thickness has been used to calculate the parameter $f=\frac{\ln2\;}{2\pi R}$ (Figure 2). Then, the electrical conductivity can be obtained as the slope of the linear relationship f=σt.

The semilog plot in the inset of Figure 2 shows that the same relationship holds over several decades. The electrical conductivity of the semiconductor material, given as the slope of the linear plot, is σ=6.67 × 10^−5^ S/m. Correspondingly, the resistivity at room temperature is ρ = 1.5 × 10^7^ Ω m, which is in agreement with ref. [21].

**Figure 3:**
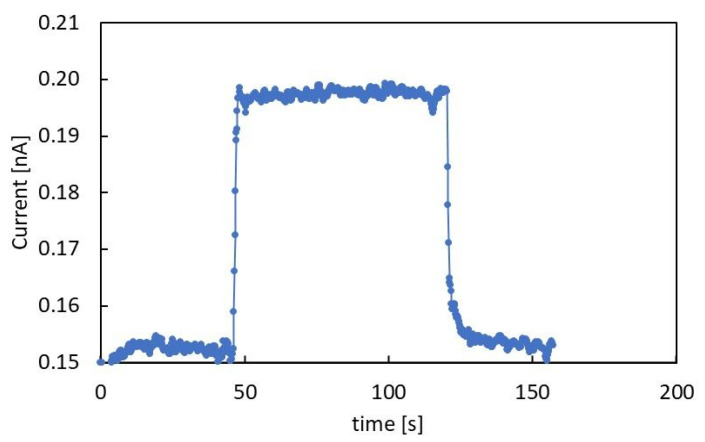
Photocurrent response of the 1.5 μm thick CsPbCl_3_ film with 400 V external voltage applied under illumination with 365 nm LED power 16.5 mW.

Figure 3 shows the photocurrent flowing in the 1.5 μm thick film, with a 400 V applied voltage, during illumination of the film with a 365 nm UV LED. The photocurrent response is characterized by fast rise and decay times and a stable photocurrent during the illumination, indicating that a stationary condition is obtained. A set of measurements has been carried out at different power at room temperature. Here, the LED power was varied in the range 0.8–22.8 mW by changing the current input in the range 50–1600 mA. In these kinds of measurements, the bias Vb was kept at 800 V, which is high enough to get a measurable photocurrent above the background value measured in dark even at the lowest power conditions.

**Figure 4:**
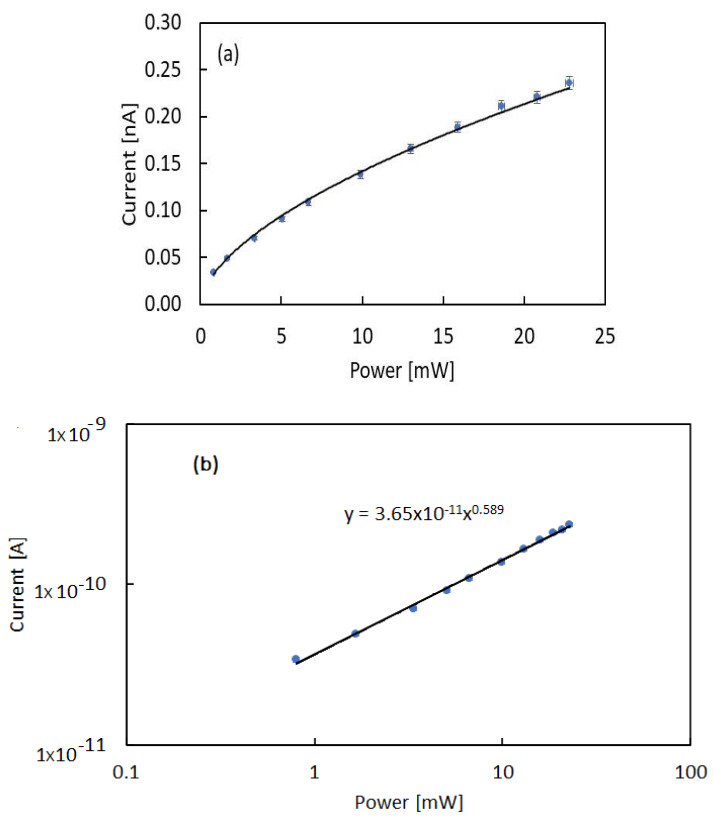
Photocurrent *I* measured with a bias Vb = 800 V at room temperature (T = 296 K) as a function of the UV LED optical power P plotted as a function of the power P in: (**a**) linear and (**b**) log–log plots. Best-fit to data evidence a power function: $I\left(P\right)\propto P^{\gamma }$ with γ ≈0.6.

Figure 4a,b show the photocurrent plateau value measured at each investigated LED power, *P*, as a function of *P*, in linear and log–log plots, respectively. Data show that the photocurrent is a sublinear function of the optical power, the log–log plot in particular evidences that a power function holds: $I\left(P\right)\propto P^{\gamma }$. Best-fit to data are obtained considering γ = 0.589.

### 3.2. Optical Characterization in the Phase Transitions Range

**Figure 5:**
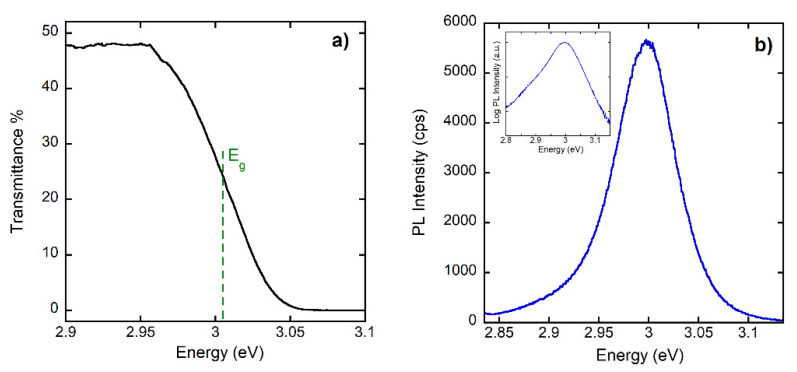
(**a**) Transmittance spectrum at room temperature. (**b**) Typical PL spectrum at room temperature. In the inset, the same spectrum is shown in log scale.

The typical RT transmittance spectrum is shown in Figure 5a, providing the value of the band gap. Figure 5b shows a typical RT PL spectrum of the 1.5 µm-thick sample. In the inset of Figure 5b, the PL spectrum shown in log scale evidences the presence of high and low-energy tails.

**Figure 6:**
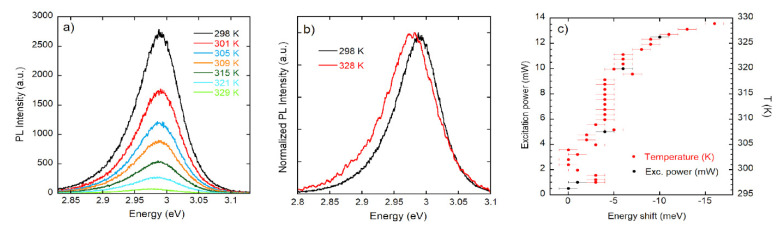
(**a**) PL spectra in the temperature range 298–329 K, (**b**) normalized PL spectra at the extreme of the explored temperature interval, (**c**) red shift of the PL peak as measured by power-dependent PL spectra at 300 K and temperature-dependent PL spectra with an excitation power of 1 mW.

PL spectra in the temperature range 298–329 K are shown in Figure 6a for the 1.5 µm thick sample. Increasing the temperature, we find a reduction of the intensity and a red shift of the PL peak energy (see Figure 6b where, for clarity, spectra have been normalized). In Figure 6c, the red shift is compared for power-dependent measurements at 300 K with the temperature-dependent shift at the excitation power of 0.1 W cm^−2^. A similar red shift is observed in Figure 6c when the excitation density is changed at room temperature and the generated electron–hole pair density is in the range 10^17^–10^20^ cm^−3^. This comparison reveals a sample heating of a few tens of degrees from the minimum to the maximum power used in the experiment. An absence of shift is observed around 315 K where phase transitions are reported for CsPbCl_3_.

### 3.3. Electrical Characterization in the Phase Transitions Temperature Range

**Figure 7:**
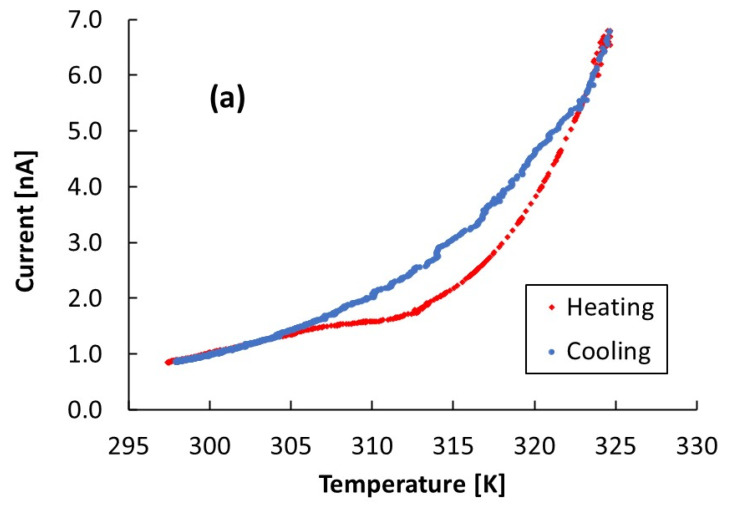

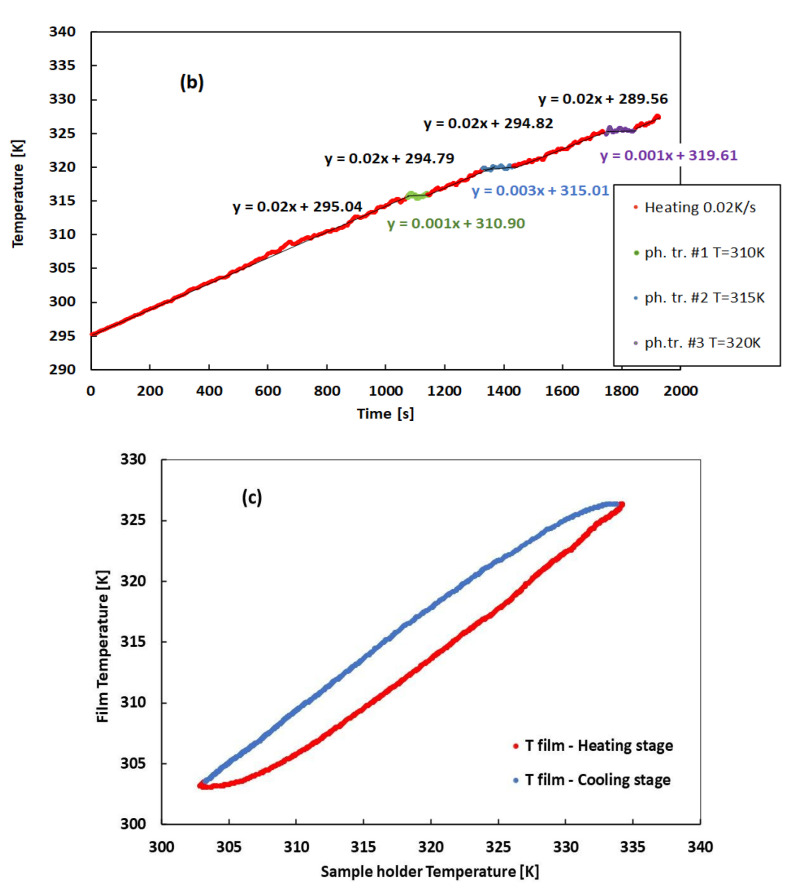
A 1.5 μm thick CsPbCl_3_ film (**a**) Current–temperature at 800 V in the temperature range 295–325 K. (**b**) Dependence of the temperature of the film with time. In both measurements, the change of temperature with a constant rate 0.02 K/s is driven by a temperature sensor close to (but not directly on) the CsPbCl_3_ film. (**c**) Temperature measured by a sensor placed on the CsPbCl_3_ film as a function of the temperature measured by the temperature sensor on the sample holder.

Figure 7a shows the behavior of the dark current of the CsPbCl_3_ 1.5 µm thick film as a function of the temperature. Here, the temperature is read out by means of a sensor placed on a glass over the sample holder, which is close to the film. The sensor drives an automatized control system used to keep the temperature rate during heating and cooling constant at 0.02 K/s. The voltage applied across the electrodes is 800 V. The cooling stage follows an exponential trend, while the heating stage, instead, shows a retard of the current in the range of temperature 305–325 K, with respect to the current measured during the cooling stage. To investigate this effect in detail, we have measured the temperature of the film by placing another temperature sensor directly on the film and comparing the measured temperature with the value provided by the sensor driving the temperature controller. Figure 7b shows the results: starting from T = 295 K, the temperature increases linearly with time with fixed slope, 0.02 K/s, up to 330 K, except in three regions, where the slope of the linear curve becomes one order of magnitude lower. Here, the temperature is almost constant, respectively, around 310 K, 315 K, and 320 K, values almost corresponding to the intercept of the linear fit in the corresponding regions. Figure 7c shows the temperature measured by a sensor placed on the CsPbCl_3_ film as a function of the temperature measured by the sensor on the sample holder in case of β = 0.1 K/s. Temperature hysteresis is indeed observed in the range 305–330 K.

**Figure 8:**
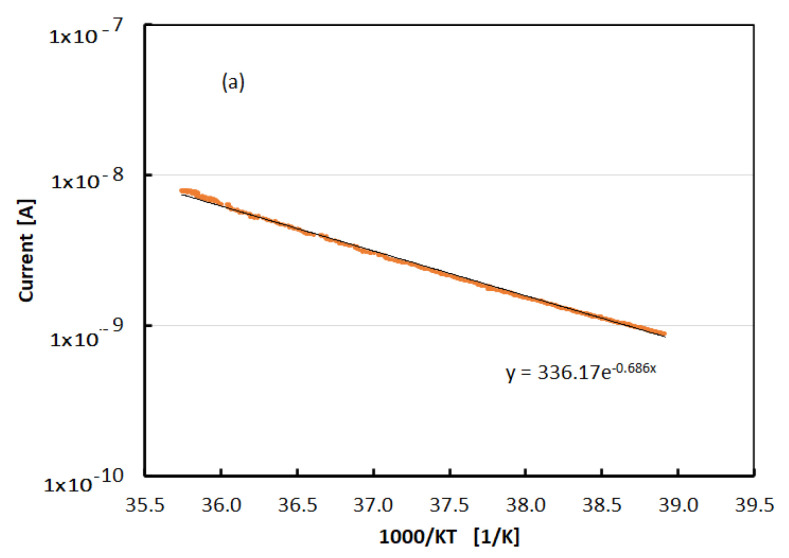

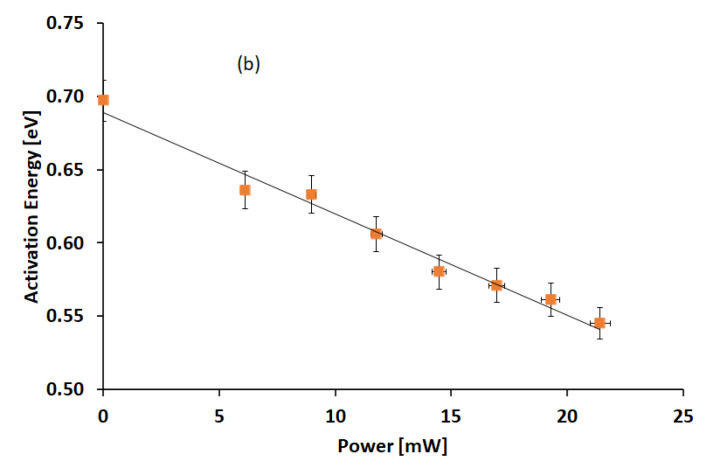
(**a**) The logarithm of the current plotted as a function of 1/KT when a fixed bias Vb = 800 V is applied across electrodes. The best-fit to data is exponential with negative slope, showing that the current is thermally activated, and no hysteresis is observed during the cycle. (**b**) Activation energy plotted as a function of the UV LED illumination power and linear best-fit.

To avoid this thermal mismatch, further measurements of current–temperature (I–T) characteristics have been carried out with the temperature sensor placed directly on the film when a fixed bias was applied across the electrodes. I–T curves have been measured in dark and under UV LED at different illumination power. A typical result is shown in the Arrhenius plot of Figure 8a in case of Vb = 800 V and UV LED illumination with P = 9 mW. No hysteresis is observed during the thermal cycle. Figure 8b shows the thermal activation energy of the current characteristics measured as the slope of the Arrhenius plot plotted as a function of the UV LED illumination power together with its linear best-fit.

**Figure 9:**
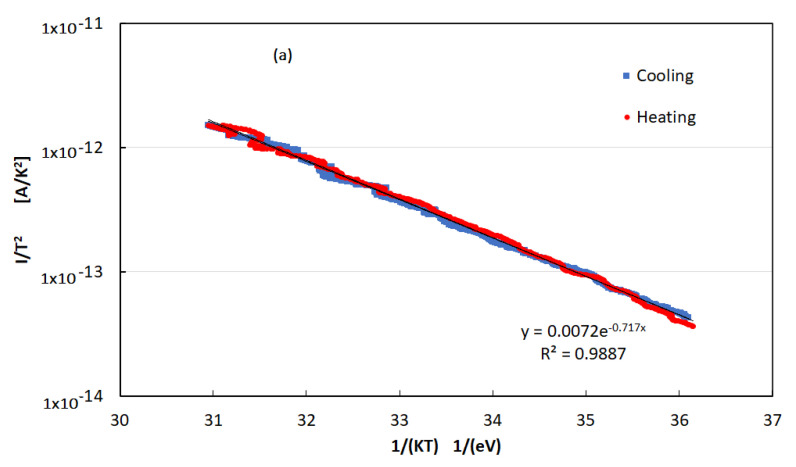

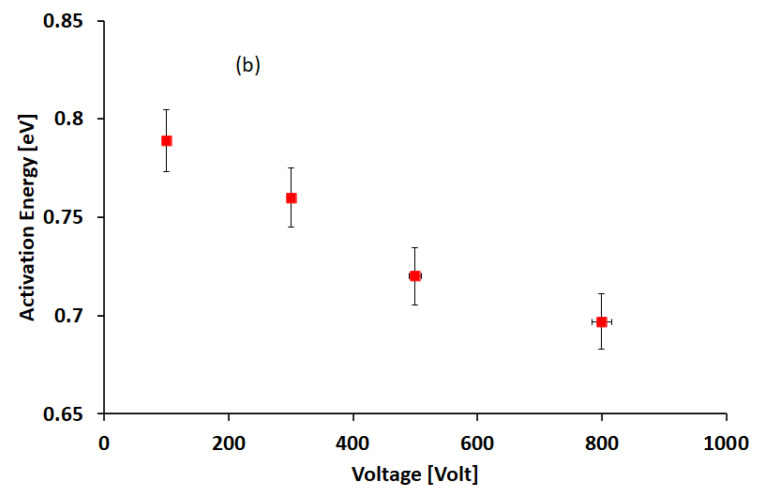
(**a**) Thermally stimulated currents measured after illumination with the 365 nm LED at room temperature, carried out in the range above the phase transitions, from 320 to 373 K, with constant rate 0.1 K/s and 500 V bias voltage. (**b**) Activation energy of the TSC curves plotted as a function of the applied bias.

We have also performed current–temperature characteristics above the phase transition range, up to 370 K, to reveal the presence of deep traps possibly influencing transport properties at room temperature. Thermally stimulated currents (TSC) have been measured in dark after illuminating the film with the UV LED for several minutes at room temperature in view to change the occupancy of deep traps. The Arrhenius plot of a TSC measurement was made with an applied bias of 500 V and $\beta =0.09\frac{K}{s}$; the constant heating rate is shown in Figure 9a. We observe no differences between the current read-out during heating and cooling stages. The activation energy of the current is E_A_ = 0.72 eV in this measurement. The activation energy value is dependent on the applied voltage, as shown in Figure 9b.

## 4. Discussion

Concerning the electrical measurement at room temperature, we observe that current transient shown in Figure 1a, mainly due to RC effects, is always limited to 10–20 s; afterwards, the current stabilizes at a value independent of the previous electrical history of the sample. This means that polarization effects within the material may be considered negligible. As a consequence, the I–V characteristics show no hysteresis, as displayed in Figure 1b. Figure 2 shows that the function f(t) obtained using the data pairs (*t, f*) is a linear function of the thickness *t*. This indicates a good homogeneity of the semiconductor material, deposited with the same growth parameters, independently of the deposition time. The *f*(*t*) function is linear with the film thickness but shows a negative intercept, indicating that we have to limit our electrical measurements to films with thickness above 100 nm. A similar effect was observed in [22] when the current was measured in situ directly during the deposition of nanostructured carbon films. The linear behavior was measured only beyond a thickness of 150 nm; this result was explained considering that the dominance of a bulk-like transport mechanism was achieved above this value, while below, transport properties should be dominated by percolation processes in a constrained geometry.

The set of measurements carried out during illumination with the 365 nm LED and different power at room temperature shown in Figure 4a evidence that the relationship between power and photocurrent is non-linear. A power function trend with a 0.6 exponent is evidenced in the log–log plot of Figure 4b. This power behavior can be described considering that photocurrent is regulated by trapping–recombination effects at defects. Starting from the simplest model with one recombination center with density *N_RC_*, the rate equation for *n*, electron density in conduction band [23], is:

(2) $$\frac{dn}{dt}=G-nc_{n}\left(N_{RC}-n_{RC}\right)$$

with $c_{n}$ as the electron capture coefficient, an electron–hole *G* generation rate, and *n_RC_* electrons trapped by the localized recombination center with energy level *E_RC_*. In stationary conditions, the relationship between *G* and *n* is:

(3) $$G=nc_{n}\left(N_{RC}-n_{RC}\right).$$

**Figure 10:**
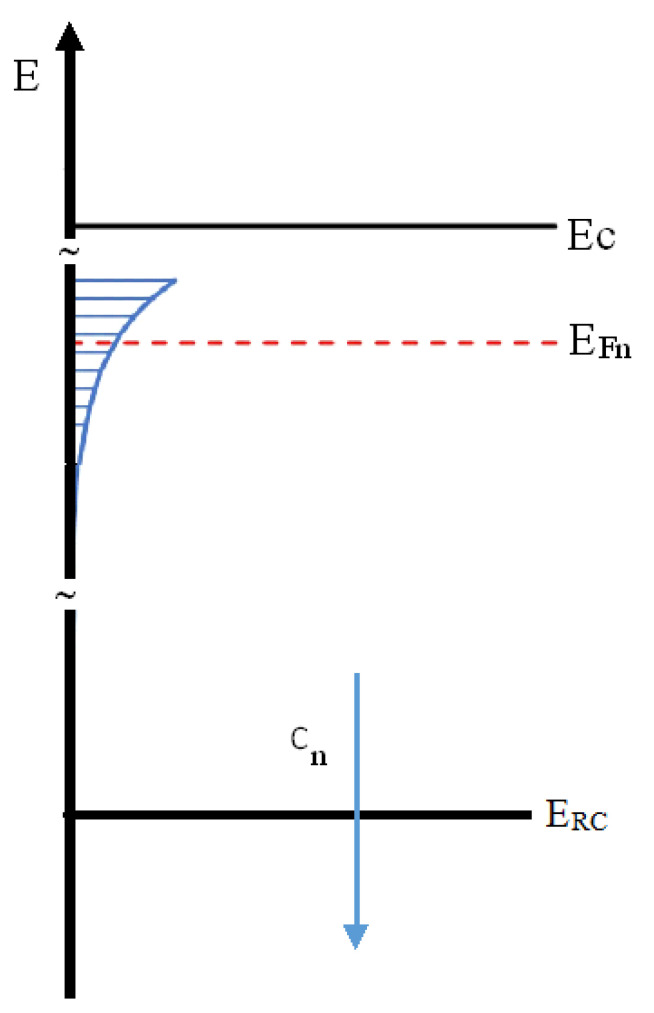
Band diagram showing that the intragap DoS has an exponential tail of defects below the conduction band edge *E_C_*: the quasi-fermi level *E_Fn_* is within the exponential trap distribution. The recombination center with capture coefficient *c_n_* is characterized by a deeper energy level at *E_RC_*.

For either small *n* values or low illumination intensity, there is a hole in a recombination center for each electron in the conduction band: $n\approx \left(N_{RC}-n_{RC}\right).$ Thus, a square root dependence holds: $n\propto G^{1/2}$. Considering the linear dependence of *G* on the power $G=\frac{\alpha P}{h\nu }$ and the photocurrent density given as: I=qvnA, with *v* drift velocity and *A* surface normal to the electric field lines, we find the sublinear relationship: $I\propto P^{1/2}$. To improve this simplistic model, we may take into consideration that lattice disorder usually adds a tail of allowed energy states within the gap below the conduction/valence band edge *E_c_*. Modeling the intragap DoS as an exponential tail, putting at *E* = 0 the border between extended and localized states (*E_c_* = 0) [24]:

(4) $$g\left(E\right)=\frac{N_{L}}{E_{L}}\;e^{\frac{E}{E_{L}\;}}$$

with *E_L_* tailing factor, then $N_{L}=\int_{-\infty }^{0}g\left(E\right)dE$ is the total density of localized states within the band gap. We may write the decay constant in the form $E_{L}=KT^{*}$, where *K* is the Boltzmann constant and $T^{*}$ is a characteristic temperature. As shown in [23], for Fermi level *E_Fn_* positioned within the intragap exponential tail (see Figure 10), T* ≥ T and in case balance $n\approx \left(N_{RC}-n_{RC}\right)$ is valid due to either low injection or small *n*, one obtains $n\propto G^{\gamma }$ with $\gamma =\frac{T^{*}}{\left(T+T^{*}\right)}$ → 0.5<γ<1.0. Using data shown in Figure 4, γ=0.5892 and T=296.15K, we have $T^{*}=424.8\;K$, and the tailing factor is $E_{L}=36.6\;\mathrm{meV}.$

Coming to the optical characterization data, we first consider the typical RT transmittance spectrum shown in Figure 5a, which provides a value of the band gap at (3.005 ± 0.005) eV. This value is in agreement with literature data [25] and with the RT PL spectrum measured for the 1.5 µm thick sample shown in Figure 5b, which peaked at (2.998 ± 0.002) eV. In the inset of Figure 5b, the PL spectrum is shown in log scale, evidencing the presence of the high-energy tail coming from the recombination of the thermalized distribution of excitons and carriers, while the low-energy tail arises from the absorption Urbach tail [26]. In Figure 6a, increasing the temperature, we observe a reduction of the intensity of roughly a factor 40 and a red shift of the PL peak energy of 7 meV. In Figure 6c, the red shift is compared for power-dependent measurements at 300 K with the temperature-dependent shift at the excitation power of 0.1 W cm^−2^. A blue shift of the band gap is usually reported in inorganic halide perovskites when the temperature changes between 10 and 300 K. To our knowledge, there is only one report [27] concerning CsPbCl_3_ above room temperature which shows an increase in the blue shift. However, the low-temperature values for the band gap energy below 300 K in ref. [27] are strongly different with all the rest of published experimental results ([12] and refs therein). The red shift reported in Figure 6a–c highlights a change in the band gap energy, which is possibly related to the phase transitions that are reported for CsPbCl_3_ in the T range (300–330 K) [13,14,15,16]. Quite remarkable is also the observation (see Figure 6c) of a similar red shift at room temperature when the excitation density is changed, the generated electron–hole pair density in the range 10^17^–10^20^ cm^−3^, and the absence of shift in the temperature range around 315 K where phase transitions are reported for CsPbCl_3_.

**Figure 11:**
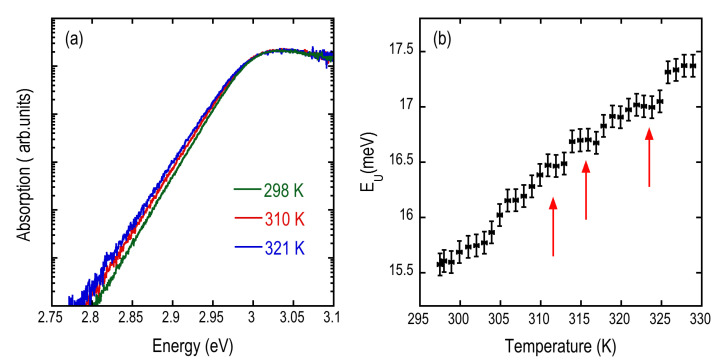
(**a**) Absorption curves as extracted from the PL spectra of Figure 6a for three temperatures. (**b**) Urbach energy E_U_ as a function of temperature as evaluated from the absorption curves.

According to the procedure used in several references (see, for instance, refs. [26,28,29]), we extracted the absorption dispersion from the PL spectra, assuming a thermalized excitons/carriers population. The results are reported in Figure 11a for three different temperatures, showing the presence of the Urbach tail over five orders of magnitude. By an exponential fit of the Urbach tail, we evaluate the Urbach energy E_U_. The dependence of E_U_ on the temperature is shown in Figure 11b where the red arrows indicate the temperatures where the phase transitions are reported in the literature [13,16,21,30]. A flattening in the Urbach energy is found in a restricted temperature interval around each phase transition: such behavior represents a significant deviation from what was found and expected, considering the origin of the Urbach tail in halide perovskites related to the dynamic disorder induced by the lattice vibration [26]. The way the experiment was performed (changing T in steps and waiting several minutes after each step in order to stabilize T) excludes that the pedestals are related to latent heat-related effects; therefore, additional contributions as strain release between the film and the substrate and within the grains in the film have to be invoked to explain the behavior of E_U_ at the phase transition temperatures.

Evidence of the phase transitions around room temperature encountered in optical measurements is also found in the electrical characterization performed as a function of the temperature. In Figure 7a, the cooling stage follows an exponential trend, according to a thermally activated current: $I\left(T\right)\propto T^{2}e^{-\frac{E_{A}}{KT}}$, with *E_A_* as the activation energy and K as the Boltzmann constant. Instead, the plot of the heating stage shows a retard of the current in the range of temperature 305–325 K with respect to the current measured during the cooling stage. The results shown in Figure 7b evidence three regions where the temperature is almost constant. This may be interpreted considering that the energy released by the heating resistance is absorbed in the form of latent heat by the film to make the phase transition occur. In this way, we may determine three phase transitions, respectively, at 310 K, 315 K, and 320 K, corresponding to the intercept of the linear fit in the corresponding regions. Figure 7c showing the temperature measured by a sensor placed on the CsPbCl_3_ film vs. the temperature measured by the sensor on the sample holder shows a temperature hysteresis in the range 305–330 K, as that shown by the current in Figure 7a. This behavior suggests that the current–temperature characteristics shown in Figure 7a are subjected during heating to the effect of latent heat due to the occurring of phase transitions. During the cooling stage, the latent heat is released directly in the atmosphere around the film, so it does not produce a significant temperature mismatch between the sample holder and film. Finally, quite remarkable is the agreement between the results of Figure 11b and those of Figure 7b.

Measurements shown in Figure 8a,b concern the evaluation of the activation energy *E_A_*_,_ in the expected expression of the current, $I\left(T\right)\propto T^{2}e^{-\frac{E_{A}}{KT}},$ in different illumination conditions. *E_A_* is calculated as the slope of the I(T)/T^2^ logarithm plotted as a function of 1/(KT), where T is measured with the temperature sensor placed on the film T. The best-fit in Figure 8a suggests the activation energy E_A_ = 0.69 eV. The best-fit to data of Figure 8b shows that the activation energy value is decreasing linearly as the power increases. We may interpret the distance of the Fermi level *E_Fn_* from the conduction band edge *E_c_* as $E_{c}-E_{Fn}\approx E_{A}$; the activation energy is measured in the I–T characteristics. In dark, at this applied voltage, we have $E_{c}-E_{Fn}=0.74\;\mathrm{eV}$. With illumination, $E_{Fn}$ is moving within the exponential tail of energy states toward the conduction band edge so the activation energy of the current is progressively lowering.

**Figure 12:**
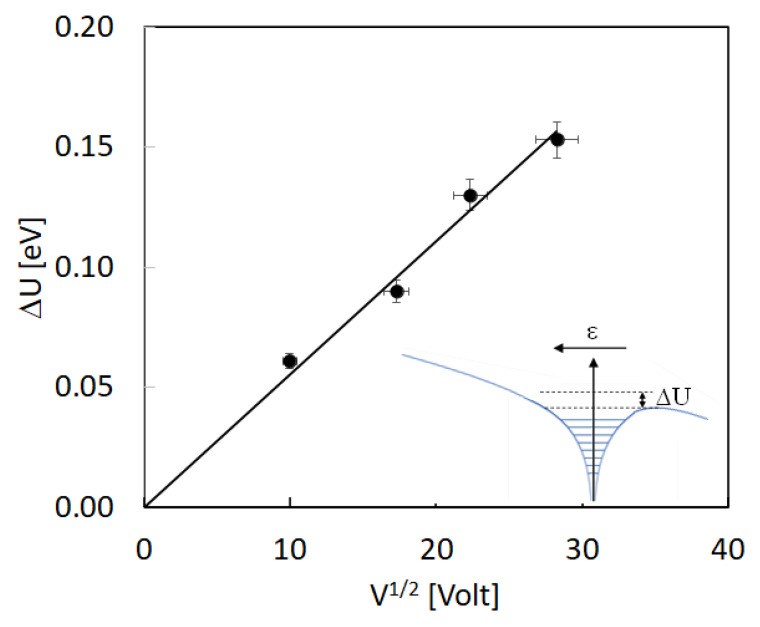
Barrier lowering due to the Poole–Frenkel effect of the energy of the trap associated to the initial rise plots of the TSC measured at different bias, plotted as a function of the square root of the applied bias.

Finally, to briefly discuss our thermally stimulated currents results, we consider the presence of deep traps, for simplicity with a constant energy level *E_t_* from the conduction band edge and capture cross section σ. Illumination of the sample at room temperature during the priming stage should change the trap occupancy. As a consequence, during the following heating scan, the charge in excess should be emitted, originating a peak in the current, which will be absent in the returning cooling stage. The TSC current may be expressed as [23,31]:

(5) $$I_{TSC}=-qlAe_{n}\left(t\right)n_{t}\left(t\right)$$

where $n_{t}$ is the concentration of traps filled with electrons, $e_{n}=N_{c}\sigma v_{th}exp\left(-\frac{E_{t}}{kT}\right)$ is the emission coefficient for electrons, $N_{C}=2{\left(\frac{2\pi m^{*}KT}{h}\right)}^{\frac{3}{2}}$ is the effective density of states in the conduction band, and $v_{th}=\sqrt{\frac{3KT}{m^{*}}}$ is the thermal velocity. The parameter *l* is a constant that may be interpreted as the average distance charge carriers travel in the electric field before recombining. Its expression is $l=\tau_{eff}\varepsilon {µ}_{n}$, where ε is the electric field, *μ_n_* is the electron mobility, and $\tau_{eff}$ is the effective collection time. In case the TSC is performed in a temperature range where the trap is not significantly changing its trap occupancy, the change between heating and cooling currents will be negligible, and we will see no peak. This situation, called the “initial rise range”, will show a dependence of the current depending only on the emission coefficient *e_n_*(*T*), as the concentration of the filled traps is not changing significantly:

(6) $$I_{TSC}\left(T\right)=-qlAe_{n}\left(T\right)n_{t}=\delta \sigma T^{2}exp(-\frac{E_{t}}{kT})$$

with $\delta =2qlA{\left(\frac{2\pi m^{*}K}{h}\right)}^{\frac{3}{2}}\sqrt{\frac{3K}{m^{*}}}$. The current will appear to be thermally activated with activation energy given by the energy *E_t_* of the trap. Nonetheless, in our measurements, we observe that the activation energy is a function of the applied bias, with values in the range 0.78–0.85 eV. This suggests the occurrence of a Poole–Frenkel effect, which is due to the electric field lowering of the trap barrier for charge emission, in case of high electric field $\varepsilon ⪆10^{5}\;V/m$. The barrier lowering absolute value, ΔU, according to the Poole–Frenkel effect should be dependent on the square root of the applied electric field [32]. This is indeed our case, as shown in plot of Figure 12. In the figure, the barrier is calculated considering an energy of the trap at zero electric field equal to *E_t_* = 0.95 eV.

We may also tentatively estimate the possible range of values of the capture cross-section of this deep trap, considering the possible occurrence of a TSC peak due to this trap above the maximum temperature investigated by us. We know that the temperature T_peak_ where the TSC peak should occur is related to the energy and the cross-section of the trap by the expression [31,33]:

(7) $$ln\;\left\{\frac{T_{max}{}^{4}}{\beta }\right\}=\frac{E_{t}}{kT}+ln\;\left(\frac{E_{t}}{\sigma \delta T_{max}}\right)\;.$$

This equation can be used to evaluate a range of possible capture cross-section values starting from *E_t_* = 0.9 eV determined with the initial rise method and considering that the peak temperature should be above the highest value investigated in this work, T = 373 K: thus, we obtain $\sigma ⪅5\cdot 10^{-22\;}m^{2}.$ Finally, we may tentatively estimate the range of concentration of filled deep traps, n_t_ from Equation (6), by evaluating the intercept of the semilog plot, $ln\;\left(\frac{I}{T^{2}}\right)\mathrm{vs}\cdot \;\frac{1}{kT}$, considering the possible range of values of the capture cross-section *σ*, hypothesizing a charge collection distance *l* equal to the distance between electrodes, 3 mm in our case, and the highest value of cross-section, $\sigma =5\cdot 10^{-22}\;m^{2}$, we get: $n_{t}≅10^{21}\;m^{-3}$. It is already known that in lead halide perovskites, defects are mainly due to halide vacancies [34]; in case of CsPbCl_3_, the Cl vacancy has been related to a deep energy level almost at midgap. The low values of the cross-section and defect density estimated in this work support that this material, as other lead halide perovskites, is indeed a defect-tolerant material.

## 5. Conclusions

We have presented a detailed experimental study concerning the electrical and optical properties of CsPbCl_3_ films prepared by radio-frequency magnetron sputtering around the phase transitions above room T. We get clear evidence of three phase transitions between 310 and 320 K which show up in a non-linear behavior of the current-temperature characteristics and in the PL shift with T. The current appears to be thermally activated with activation energy in the range 0.6–0.8 eV. The dependence of the activation energy with the optical power has been explained with the presence of an exponential density of intragap levels. The presence of deep traps has been revealed through an initial-rise study of the thermally stimulated currents. Our results provide insights on trap parameters as activation energy and their cross-sections.

## Data Availability

All data are contained within the article.
